# Assessment of Impact of Containment During the COVID-19 Epidemic and Coping Behaviours Using Newly Developed Assessment Tools

**DOI:** 10.3389/fpubh.2021.787672

**Published:** 2021-12-22

**Authors:** Li Ping Wong, Haridah Alias, Mahmoud Danaee, Hai Yen Lee, Kit Mun Tan, Peter Seah Keng Tok, Mustakiza Muslimin, Sazaly AbuBakar, Yulan Lin, Zhijian Hu

**Affiliations:** ^1^Faculty of Medicine, Department of Social and Preventive Medicine, Centre for Epidemiology and Evidence-Based Practice, University of Malaya, Kuala Lumpur, Malaysia; ^2^Department of Epidemiology and Health Statistics, School of Public Health, Fujian Medical University, Fuzhou, China; ^3^Faculty of Medicine, Department of Medicine, Centre for Epidemiology and Evidence-Based Practice, University of Malaya, Kuala Lumpur, Malaysia; ^4^Institute for Clinical Research, National Institutes of Health (NIH), Ministry of Health Malaysia, Shah Alam, Malaysia; ^5^Faculty of Health Sciences, Department of Medical Science & Technology, PICOMS International University College, Kuala Lumpur, Malaysia; ^6^Faculty of Medicine, Department of Medical Microbiology, University of Malaya, Kuala Lumpur, Malaysia

**Keywords:** psychological, confinement measures, COVID-19, partial least squares, exploratory factor analysis

## Abstract

**Background:** The confinement measures during COVID-19 had a massive effect on physical and psychological health in public. This study assessed the impact of containment and coping behaviour among the Malaysia public during the COVID-19 pandemic. Questions assessing the impact of containment and coping behaviours were developed and psychometrically tested.

**Methods:** Exploratory factor analysis (EFA) was conducted with the items using principal component analysis extraction and Varimax rotation. Partial least squares structural equation modelling was used to determine the relationship between coping and impact.

**Results:** The 13-item of impact and 10-item coping instruments were developed with three dimensions identified through EFA. Both scales demonstrated excellent composite reliability and good convergent validity. The survey findings revealed that the impact on individual psychological aspects was prominent, followed by well-being and lifestyle. Mindfulness and physical coping strategies were most commonly reported. Coping through seeking help from health professionals and hotlines had a positive direct effect on well-being and lifestyle (b = 0.231, *p* < 0.001), psychological (B = 0.132, *p* < 0.001), and employment-related (0.194, *p* < 0.001) impacts. Coping through mindfulness practise had a negative effect on well-being and lifestyle-related impact (B = −0.180, *p* < 0.001) and employment-related impact (B = −0.096, *p* = 0.008).

**Conclusions:** Despite some limitation, the scales for measuring impact and coping behaviours have the potential to be used as a measurement tool in future studies. Findings highlight the enormous impact of the pandemic on psychological well-being and lifestyles. Health authorities should support individual coping as it was found to be an important resilience-related factor to mitigate the impacts of containment during the pandemic.

## Introduction

The 2019 coronavirus disease (COVID-19) outbreak, which began in Wuhan, China, in December, has become a global health challenge and resulted in significant morbidity and mortality. Worldwide SARS-CoV-2 infections topped 20 million as of mid-August 2020 (1). The rapid increase in COVID-19 cases has prompted many governments around the world to introduce confinement measures to contain the epidemic. These measures have led to many businesses being shut down temporarily and a reduced workforce across all economic sectors. Along with its high infectivity and fatality rates, containment during the COVID-19 pandemic has imposed a universal economic burden and financial losses. The confinement measures have also had a massive effect on physical and psychological health (2, 3). People have become suddenly inactive and adopted sedentary behaviours, resulting in an unprecedented health crisis as self-isolation and living in confinement for several weeks to months represents a physiological challenge with significant health risks, especially in people with chronic diseases (4, 5). With respect to psychological health, the high contagiousness and fatality rates provoke fear, anxiety, and depression in the public, which results in increased mental issues in society (6, 7). Further stigma and discrimination are other aspects of the outbreak of the pandemic that add to the psychological health burden (8).

As in many countries around the world, Malaysia, a country in Southeast Asia, is also significantly impacted by the COVID-19 pandemic. Malaysia announced the first three cases of COVID-19 on 25 January 2020. Subsequently, the country implemented a nationwide movement control order (MCO) to curb the outbreak on 18 March 2020. The MCO order included the closure of schools and higher education institutions, “non-essential” businesses, as well as a general prohibition of mass movements and gatherings across the country including religious, sports, social, and cultural activities. The public has been asked to engage in social distancing, self-isolation and in-home confinement. During the MCO, only one person was allowed to represent a household to perform necessary tasks and errands. Over the MCO period, the public was concerned with the uncertainty over how long the COVID-19 pandemic will persist. Malaysia has gone through four MCO phases, each phase lasting 2 weeks. A Conditional Movement Control Order (CMCO) was implemented from 13 May to 9 June, and a Recovery Movement Control Order (RMCO) took effect from 10 June and will last until 31 August with more lenient restrictions.

Currently, the coronavirus pandemic is far from over in Malaysia, as well as many other countries in Asia and worldwide, and the pandemic continues to evolve rapidly. In the context of the present evolving COVID-19 pandemic, there is a need to investigate the impact as well as the coping behaviours of the public in order to help design interventions to better support the general public, should there be the resurgence of the outbreak and the re-enforcement of movement restrictions. To date, there are some knowledge gaps in the current literature with regard to the impact on and coping strategies of the Malaysian public during the MCO. An earlier study found that the level of anxiety, as well as the financial and employment impact among the Malaysian public, increased along with the duration of movement confinement (9). Nonetheless, other health and general well-being consequences of movement confinement remain unclear and have never been comprehensively reported in Malaysia. Previous international studies have shown that quarantine or confinement to contain the COVID-19 outbreak have profoundly affected the general well-being, health, and employment of the community (3, 10). The implication of such an unprecedented disruption to Malaysian society needs to be assessed empirically so that support can be provided to mitigate stressors and promote healthy behaviours.

Coping responses are expected during a pandemic and understanding individualised ways of coping in such a situation is of paramount importance (11). It is an immediate research priority to understand how the public can be supported to optimise coping strategies to mitigate their impact, and subsequently facilitate the implementation of preventive interventions in the future. During the COVID-19 pandemic, it is crucial that the public is well-supported during in-home containment, with minimal consequences on health, well-being, and economic aspects. Hence, there is also a need to investigate the resilience of the public during the pandemic period to identify coping behaviours that can effectively reduce their impact.

For these reasons, this study aimed to determine the impact on and coping behaviours of the public during the MCO period of the COVID-19 pandemic in Malaysia. To date, no standard tools are available to measure impact and coping during the COVID-19 pandemic. Thus, a questionnaire on impact and coping was developed by the research team. The psychometric testing of the impact and coping items were conducted. Secondly, the study explored the use of various coping behaviours on its implication on different components of impact.

## Methods

### Measurement Development

Questions measuring impact and coping behaviours were developed in English and then translated into Bahasa Malaysia, the national language of Malaysia. Forward and backward translation was carried out to maintain the equivalence of the questionnaire in both languages. Questions were presented in both English and Bahasa Malaysia in the survey link. Questions were first developed by the research team members. Panel experts that consist of academicians and researchers were invited to performed face and qualitative content validation of the items. The authors met to discuss the evaluations and comments from the expert panel members, including the expert panel members' suggestions for improvements. Subsequently, pilot testing was performed on 30 participants to assess the clarity of the items. Minor revisions were made and the questionnaire was further pre-tested before field administration.

The developed questions measuring impact and coping behaviours are shown in Appendix 1. The impact of COVID-19 was measured using a 13-item questionnaire that queried participants of various impacts including general well-being, lifestyle, mental, and employment aspects. The response options were scored on a three-point Likert scale: 2, *extremely*; 1, *moderately*; 0, *never*. The possible impact score ranged from 0 to 26, with higher scores representing higher levels of impact. Coping behaviours were measured using a 10-item questionnaire assessing physical and psychological coping as well as help-seeking. The response options were scored on a three-point Likert scale: 2, *most of the time*; 1, *sometimes*; 0, *never*. The possible total coping behaviour score ranged from 0 to 20, with higher scores representing greater coping difficulty.

### Survey Administration

**Figure 1:**
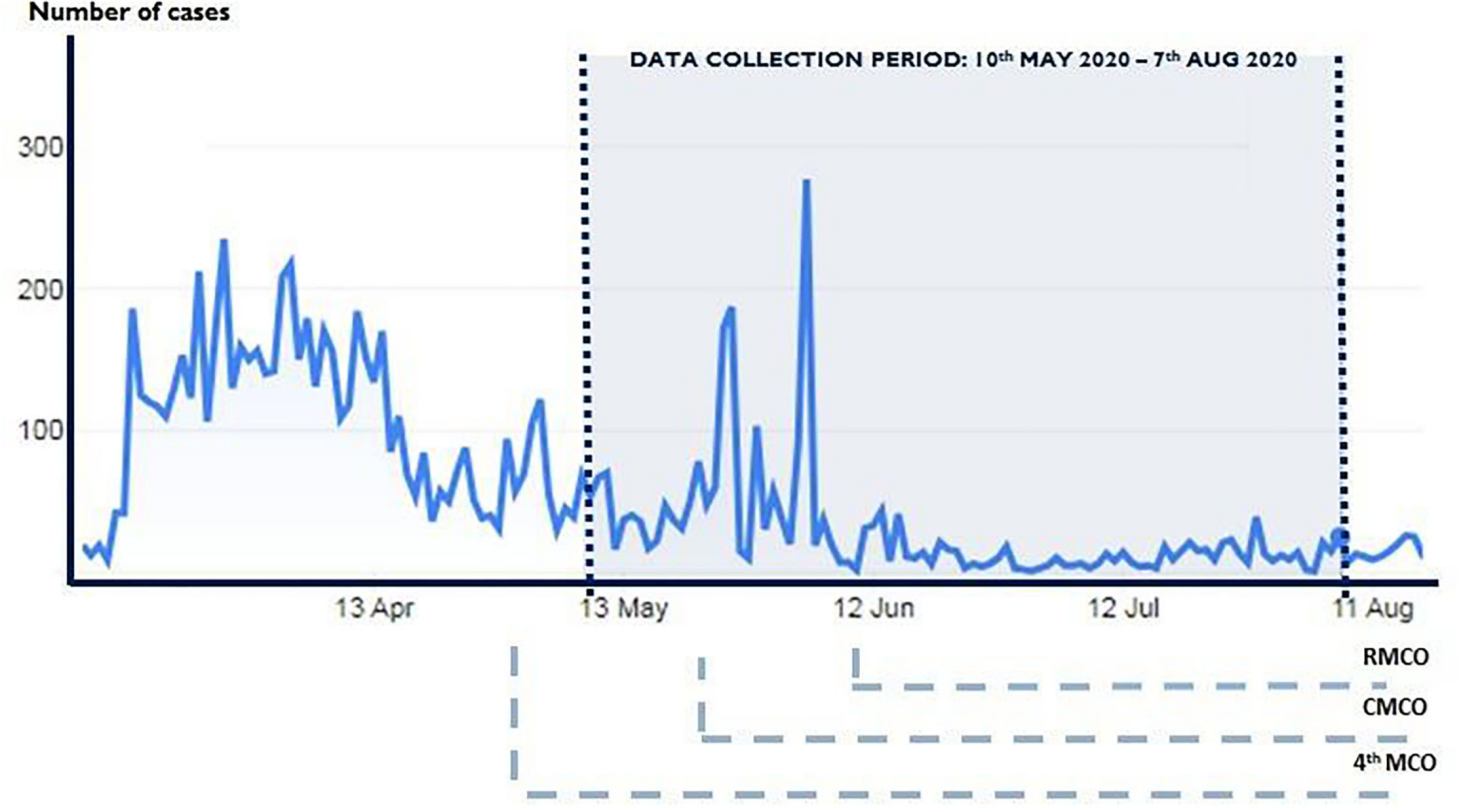
The trend of number of daily new cases in Malaysia and the survey period.

The developed measurements were administered on a diverse national sample across Malaysia. An anonymous Internet-based, cross-sectional survey was conducted between 10 May and 7 July 2020. Figure 1 shows the trend of the number of daily new cases in Malaysia from the beginning of the COVID-19 outbreak and the survey period. Snowball and convenience sampling were used to recruit the participants. The researchers used social network platforms (WhatsApp, Facebook, and Instagram) to disseminate and advertise the survey link. Respondents who completed the survey received a note to encourage them to disseminate the survey link to all their contacts. All respondents were informed that their participation was voluntary and consent was implied through their completion of the questionnaire. The survey also gathered demographic background, experience with COVID-19, and the health status of the participants. Personal details, including age, gender, ethnicity, religion, marital status, occupation, and average monthly household income were collected. The participants were also asked if they had existing chronic diseases and to rate their overall perceived health status. Overall perceived health is a subjective, individualised self-assessment of the current overall state of personal health and was measured by a single question asking for a rating of current general health status using five-item choices (“very good,” “good,” “fair,” “poor,” or “very poor”). COVID-19 experience asked participants if they knew of friends, neighbours, or colleagues who had been infected with COVID-19.

### Statistical Analysis

Descriptive statistical analyses were performed using the Statistical Package for the Social Sciences, version 25.0 (IBM Corp., Armonk, NY, USA). The significance level was set at α < 0.05, and all tests were two-tailed. The categorical data were presented as numbers and percentages. The scores for impact and coping were not normally distributed, hence are presented as medians and interquartile ranges (IQRs). The non-parametric Kruskal-Wallis and Mann-Whitney *U* tests were applied to compare the impact and coping scores between two or more groups.

The reliability of the impact and coping measurements were tested for Cronbach's alpha reliability coefficient and composite reliability. Convergent and discriminant validity was evaluated using average variance extracted (AVE) and heterotrait-monotrait (HTMT) ratio of correlations method, respectively. The Kaiser-Meyer Olkin (KMO) measure of sampling adequacy and Bartlett's test of sphericity were used to examine the appropriateness of factor analysis. Exploratory factor analysis was performed using the principal components method with Varimax rotation to determine the factor structure of the 13-item impact and 10-item coping scales. Varimax rotation maximises within-factor variance of the loadings of the factors extracted (12) and hence is preferred. Partial least squares structural equation modelling (PLS-SEM) was used to explore the association between impact and coping. This technique assesses the reliability of the dataset and the statistical significance of the coefficients and the error of the estimated path coefficients (13). The bootstrapped significance calculation was performed in SmartPLS software version 3.2.8 (SmartPLS GmbH) (14).

### Ethical Considerations

All respondents were informed that their participation was voluntary and provided consent online. This research was approved by the University of Malaya Research Ethics Committee (UM.TNC2/UMREC - 922). This study was conducted in accordance with the principles of the Declaration of Helsinki.

## Results

### Psychometric Testing of the Impact Questionnaire

Bartlett's test of sphericity yielded a significant chi-square statistic, indicating that a relationship exists between at least some of the subscales [$\chi_{\left(78\right)}^{2}$ = 3465.51, *p* < 0.05]. The analysis also produced a Kaiser Meyer Olkin (KMO) value of 0.842, indicating satisfactory sample adequacy. The communality values of the 13 items were above 0.5. Factor loading for all items was also above 0.5. Factor analysis extracted three components with an eigenvalue greater than one. The first component (6 items) explained 20.79% of the variance. The second component (4 items) and third component (3 items) explained 16.40 and 15.69% of the variance, respectively (Table 1). The three impact components were found to fit together conceptually and were named (1) lifestyle, (2) psychological, and (3) employment-related impacts.

**Table 1:** Factor loadings based principal component analysis with Varimax rotation for items related to impact and coping scales.

**Impact items**	**Component 1**	**Component 2**	**Component 3**
	**Well-being and**	**Psychological**	**Employment-**
	**lifestyle**	**psychological**	**related**
D8	0.717		
D5	0.702		
D6	0.690		
D4	0.626		
D10	0.577		
D9	0.407		
D12		0.793	
D11		0.730	
D13		0.708	
D7		0.459	
D2			0.882
D1			0.835
D3			0.528
**Eigenvalues**	2.703	2.132	2.039
**% of variance**	20.795	16.404	15.688
**Coping item**	**Component 1**	**Component 2**	**Component 3**
	**Mindfulness practice**	**Physical coping**	**Help seeking**
E3	0.796		
E1	0.735		
E4	0.726		
E2	0.71		
E7		0.802	
E6		0.738	
E5		0.623	
E8		0.581	
E10			0.93
E9			0.918
**Eigenvalues**	2.483	2.095	1.821
**% of variance**	24.825	20.949	18.207

### Psychometric Testing of Coping Questionnaire

Bartlett's test of sphericity also yielded a significant chi-square statistic, indicating that a relationship exists between at least some of the subscales [$\chi_{\left(45\right)}^{2}$ = 3308.167, *p* < 0.05]. The KMO value was 0.085. The communality values of the 10 items and factor loading were also above 0.5. Likewise, factor analysis extracted three components with an eigenvalue greater than one. The first component (4 items) explained 28.83% of the variance. The second component (4 items) and third component (2 items) explained 20.95 and 18.21% of the variance, respectively (Table 1). Similarly, the three coping behaviour components were found to fit together conceptually and were named (1) mindfulness practise, (2) psychological, and (3) help-seeking coping.

### Participant Demographics

A total of 1,052 complete responses were received. The demographic characteristics of the study participants are shown in the first and second columns of Table 2.The majority of the study participants were between the ages of 18 and 30 years old (52.9%). The proportion of female (73.1%) participants in this study was higher than males (26.9%). The majority of the study participants had a tertiary education (91.6%). By occupation category, near half were in professional and managerial occupations (46.6%), while general workers and students comprised 13.8 and 28.2%, respectively. Of the overall participants, 38.1% reported an average monthly household income of < MYR3000m while 29.1% reported an average monthly household income of MYR3001-6000. The majority of participants were from urban (66.1%) and sub-urban (23.3%) areas. Slightly over half (56.0%) of the study participants were from the central region. Only a total of 1.6% (*n* = 17) reported having a close family member infected with COVID-19. A higher proportion (16.0%, *n* = 168) reported knowing of friends, neighbours, or colleagues infected with COVID-19. The majority (92.7%) did not have any chronic diseases. The majority perceived their overall health as good (56.9%) or very good (25.2%).

**Table 2:** Demographic characteristic of study participants, COVID-19 coping and impact scores (*N* = 1,052).

**Covariates**	***N*** **(%)**	**Impact score median (IQR)**	***P*** **-value**	**Coping score median (IQR)**	***P*** **-value**
**Socio demography**
**Age group (years)**					
18–30	557 (52.9)	8.0 (5.0–12.0)		12.0 (9.0–15.0)	
31–40	272 (25.9)	7.0 (5.0–11.0)	0.009^k^	12.0 (9.0–15.0)	0.020^k^
>40	223 (21.2)	7.0 (4.0–11.0)		13.0 (10.0–15.0)	
**Gender**					
Male	283 (26.9)	8.0 (5.0–12.0)	0.22^m^	12.0 (9.0–15.0)	0.67^m^
Female	769 (73.1)	8.0 (5.0–11.0)		12.0 (10.0–15.0)	
**Marital status**					
Single	649 (61.7)	8.0 (5.0–12.0)	0.015^m^	12.0 (10.0–15.0)	0.053^m^
Married	403 (38.3)	7.0 (4.0–11.0)		13.0 (10.0–15.5)	
**Ethnicity**					
Malay	601 (57.1)	8.0 (5.0–12.0)		13.0 (10.0–16.0)	
Chinese	330 (31.4)	7.0 (4.0–11.0)	0.19^k^	12.0 (9.0–14.0)	0.001^k^
Indian	63 (6.0)	9.0 (5.0–11.0)		13.0 (9.5–16.0)	
Indigenous Sabah/Sarawak/Others	58 (5.5)	7.5 (5.0–12.0)		12.0 (10.0–15.0)	
**Highest education level**					
Secondary and below	88 (8.4)	9.0 (5.0–14.0)	0.033^m^	12.0 (9.0–15.5)	0.99^m^
Tertiary^†^	964 (91.6)	8.0 (5.0–11.0)		12.0 (10.0–15.0)	
**Occupation type**					
Professional and managerial	490 (46.6)	7.0 (4.0–11.0)		13.0 (10.0–15.0)	
General worker	145 (13.8)	10.0 (6.0–13.0)		12.0 (9.0–15.0)	
Student	297 (28.2)	8.0 (5.0–12.0)	*p* <0.001^k^	12.0 (10.0–15.0)	0.089^k^
Retired/Unemployed/Housewife	120 (11.4)	9.0 (5.0–13.0)		12.0 (10.0–15.0)	
**Average monthly household income (MYR)** ^††^					
3,000 and below	401 (38.1)	9.0 (6.0–13.0)		12.0 (10.0–15.0)	
3,001–6,000	306 (29.1)	8.0 (5.0–11.0)	*p* <0.001^k^	12.0 (10.0–15.0)	0.28^k^
6,001 and above	345 (32.8)	6.0 (4.0–10.0)		12.0 (9.0–15.0)	
**Locality**					
Urban	695 (66.1)	8.0 (5.0–11.0)		12.0 (10.0–15.0)	
Sub-urban	245 (23.3)	7.0 (5.0–11.0)	0.21^k^	12.0 (10.0–15.0)	0.73^k^
Rural	112 (10.6)	9.0 (5.0–13.0)		13.0 (10.0–15.0)	
**Region**					
Northern	147 (14.0)	8.0 (5.0–13.0)		12.0 (9.0–15.0)	
Southern	163 (15.5)	8.0 (5.0–11.0)		12.0 (9.0–15.0)	
East coast	100 (9.5)	7.0 (4.5–11.0)	0.74^k^	14.0 (11.5–16.0)	0.002^k^
Central	589 (56.0)	8.0 (5.0–11.0)		12.0 (9.0–15.0)	
Borneo island	53 (5.0)	8.0 (6.0–10.0)		12.0 (11.0–15.0)	
**Experience with COVID-19**					
**Had close family members infected by COVID-19**					
Yes	17 (1.6)	9.0 (4.0–10.0)	0.95^m^	11.0 (10.0–15.0)	0.87^m^
No	1,035 (98.4)	8.0 (5.0–12.0)		12.0 (10.0–15.0)	
**Known any friends, neighbor or colleagues infected by COVID-19**					
Yes	168 (16.0)	8.0 (5.0–11.0)	0.97^m^	12.0 (10.0–15.0)	0.61^m^
No	884 (84.0)	8.0 (5.0–12.0)		12.0 (10.0–15.0)	
**Health status**					
**Have an existing chronic disease**					
Yes	77 (7.3)	8.0 (5.0–12.0)	0.59^m^	12.0 (10.0–15.0)	0.80^m^
No	975 (92.7)	8.0 (5.0–11.0)		12.0 (10.0–15.0)	
**Perceived overall health**					
Very poor/Poor/Fair	188 (17.9)	9.0 (6.0–13.0)		11.0 (8.0–14.0)	
Good	599 (56.9)	8.0 (5.0–11.0)	0.001^k^	12.0 (10.0–15.0)	*p* <0.001^k^
Very good	265 (25.2)	7.0 (5.0–11.0)		14.0 (11.0–16.0)	

k *Kruskal-Wallis test*.

m *Mann-Whitney U test*.

† *Post-secondary education received at universities, polytechnics and colleges*.

†† *1 MYR = 0.24 USD*.

### Impact of COVID-19

**Figure 2:**
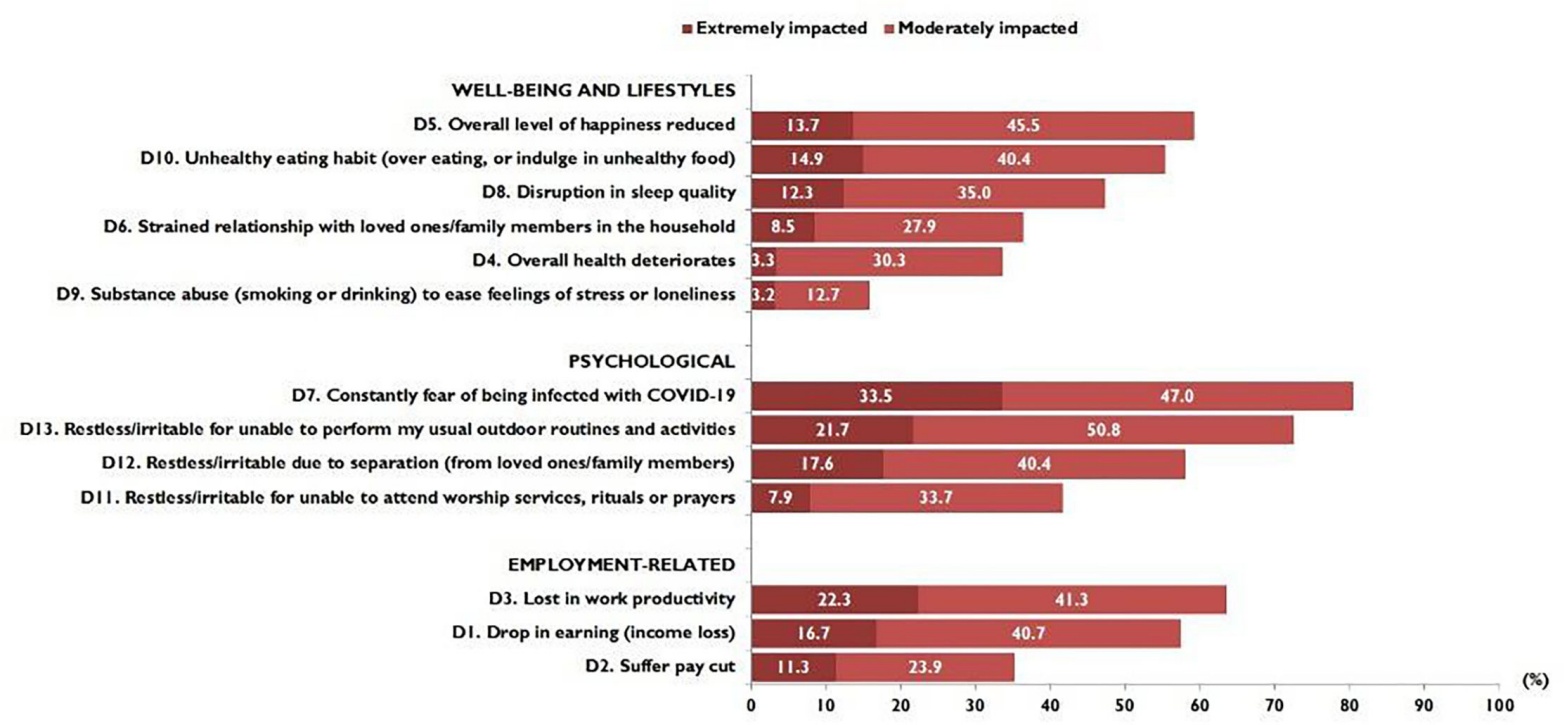
Proportion of responses on COVID-19 impact (*N* = 1,052).

Figure 2 shows the proportion of responses on the impact of COVID-19 in terms of the well-being and lifestyle, psychological, and employment-related dimensions. Overall, participants demonstrated high rates of psychological impact. The highest proportion reported being constantly in fear of being infected with COVID 19, constantly fearful or irritable over not being able to perform their usual routines, and separated from loved ones/family members. Under the well-being and lifestyle dimension, a large proportion reported an overall lower level of happiness and indulged in unhealthy eating habits. Regarding employment-related impact, a large proportion reported lower work productivity and income loss.

Of the possible maximum score of 26, the median (IQR) was 8.0 (IQR 5.0 to 11.8). Table 3 shows that participants aged 18–30 years showed a significantly greater median impact scores than the older age groups. There were no significant differences in median impact scores by gender and ethnicity. However, participants who were single reported significantly higher median impact scores than married participants. Participants with an educational level of secondary school and below reported significantly higher median impact scores (median score 9.0; IQR 5.0–14.0) than those with a tertiary education. By occupation category, participants who were general workers reported the highest median impact scores (median score 10.0; IQR 6.0–13.0). A significant inverse association was seen between median impact scores and average household income. There was a gradual decline in median impact scores as income level increased. Participants who perceived their overall health status as very poor, poor, or fair reported the highest median impact scores (median score 9.0; IQR 6.0–13.0).

**Table 3:** Results of Cronbach's alpha, composite reliability and average variance extracted.

**Construct**	**Cronbach's**	**Composite**	**Average variance**
	**alpha**	**reliability**	**extracted (AVE)**
**Impact**			
Well-being and lifestyle	0.763	0.831	0.452
Psychological	0.679	0.797	0.504
Employment-related	0.715	0.841	0.640
**Coping**			
Mindfulness practice	0.772	0.837	0.566
Physical coping	0.672	0.743	0.514
Help-seeking	0.866	0.937	0.882

### Coping Behaviours

**Figure 3:**
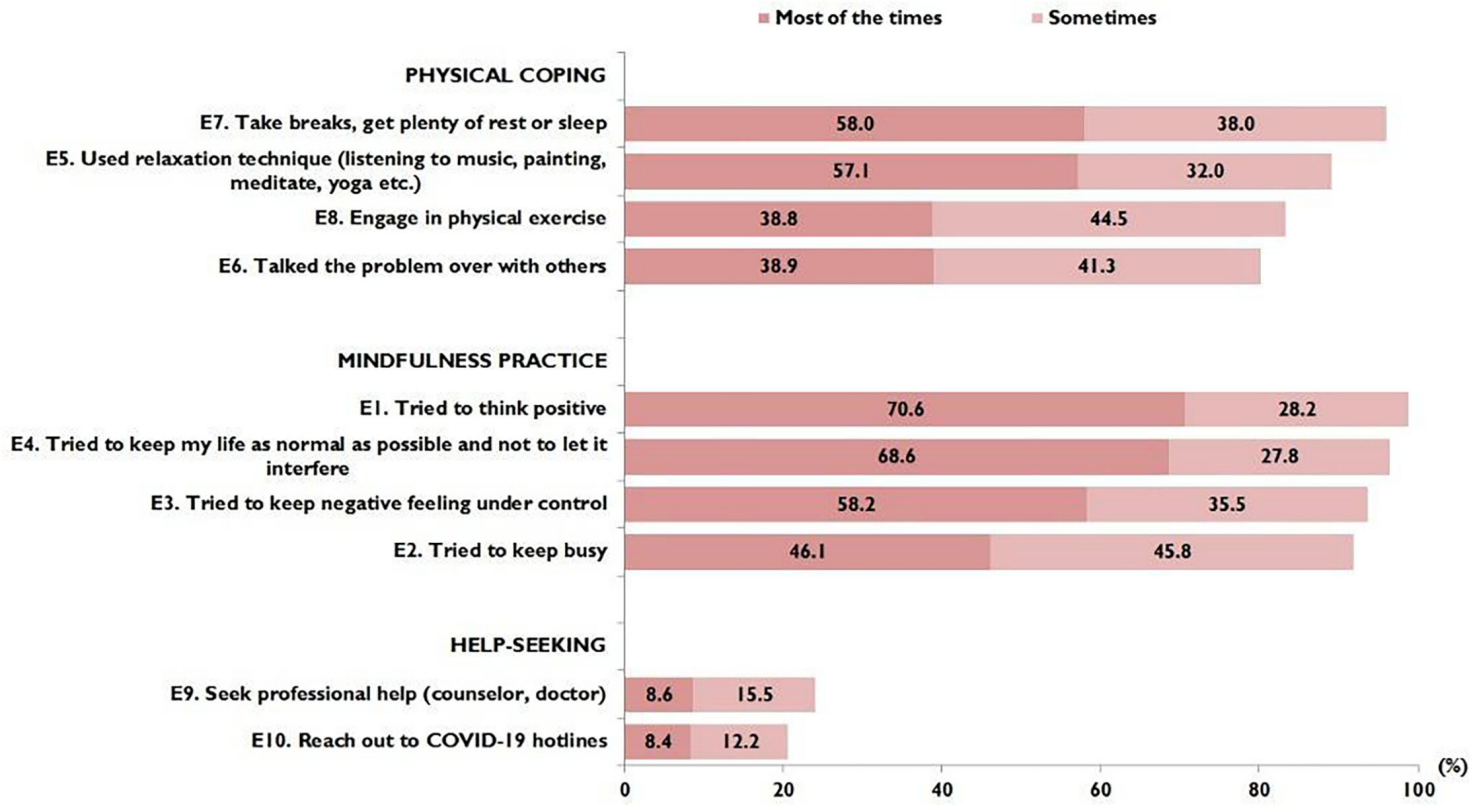
Proportion of responses on coping behaviors (*N* = 1,052).

Figure 3 shows the proportion of responses regarding coping behaviours. Mindfulness coping was most commonly reported by the study participants, followed by physical coping. Only a small proportion reported seeking professional help (23.5%) or reaching out to COVID-19 hotlines (20.6%). Of the possible maximum score of 20, the median (IQR) was 12.0 (IQR 10.0 to 15.0). Participants in the oldest age group reported significantly higher median coping scores (median score 13.0; IQR 10.0–15.0). By ethnicity, the Malay (median score 13.0; IQR 10.0–16.0) and Indian (median score 13.0; IQR 9.5–16.0) ethnic groups reported higher median coping scores, while by region, those from east coast reported higher coping scores (median score 14.0; IQR 11.5–16.0). Participants who perceived their overall health status as very good reported the highest median coping scores (median score 14.0; IQR 11.0–16.0).

### Relationship Between Coping and Impact

Table 3 shows all the results for testing the reliability of the measurement models. The results of the measurement model indicate that all the values of composite reliability (which ranged from 0.743 to 0.937) were >0.70, indicating acceptable construct reliability. Further, the Cronbach's alpha value higher than 0.6 indicates that the constructs have an acceptable level of internal consistency. Meanwhile, convergent validity, evaluated by AVE for all constructs, was >0.5 (except for well-being and lifestyle impact, AVE = 0.452). However, according to Hair et al. (15), AVE> 0.4 indicates adequate convergent validity. The discriminant validity assessment through HTMT ratio of correlations method also indicated that all HTMT values were lower than the most restrictive threshold (0.85) proposed by Kline (16), thus indicating adequate discriminant validity.

**Figure 4:**
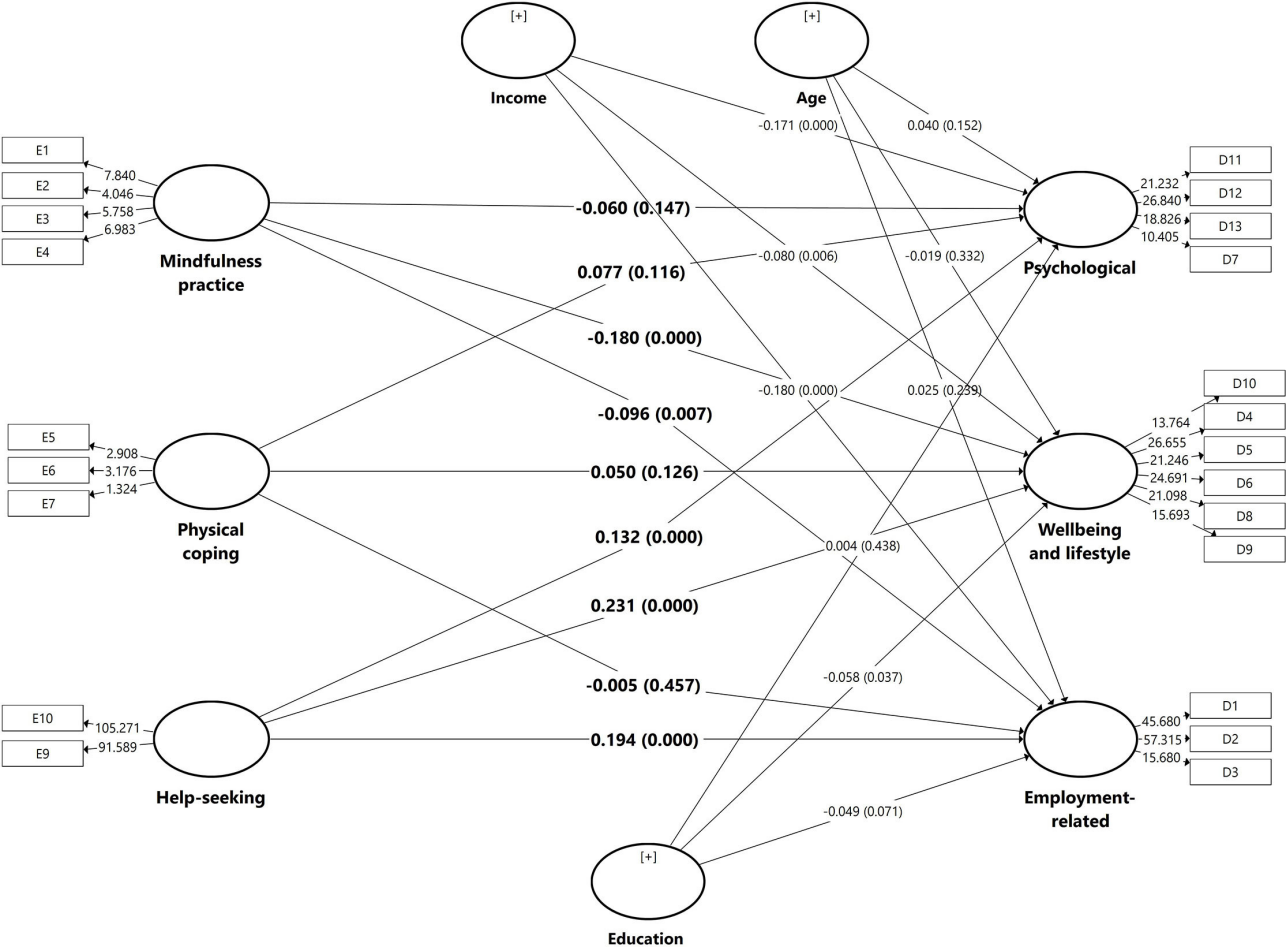
Path coefficients of the structural models predicting psychological, well-being and lifestyles, and employment related impacts.

The PLS-SEM in Figure 4 shows the associations between all the components of coping and impact. The PLS-SEM path model predicting psychological impact shows that help-seeking coping has a direct and significant effect on psychological impact (B = 0.132; *p* < 0.001). An inverse association between income and psychological impact was observed (B = −0.171; *p* < 0.001). The adjusted R^2^ value for the structural model is 0.055, showing that the model explained 5.5% of the total variance in psychological impact.

The PLS-SEM path model predicting well-being and lifestyle impact showed that help-seeking coping has a direct and significant effect on well-being and lifestyle impact (B = 0.231; *p* < 0.001). Mindfulness practise and a well-being and lifestyle impact were inversely associated (B = −0.180; *p* < 0.001). Likewise, an inverse association between income (B = −0.080; *p* < 0.001) and education level (B = −0.058; *p* = 0.037) with well-being and lifestyle impact was observed. The adjusted *R*^2^ indicates this model explained 9.5% of the total variance in the well-being and lifestyle impact.

The model for employment-related impact showed that help-seeking coping was significantly associated with higher employment-related impact (B = 0.194; *p* < 0.001). Mindfulness practise was significantly associated with higher employment-related impact (B = −0.096; *p* = 0.080). An inverse association was observed between income (B = −0.180; *p* < 0.001) and employment-related impact. The adjusted *R*^2^ indicates that the total variance explained by the model was 8.4%.

## Discussion

We queried the Malaysian public about the impact of the COVID-19 pandemic and their coping behaviours during the implementation of MCO to combat the coronavirus outbreak using self-developed instruments. The EFA extracted three components each for the measurement of impact and coping behaviours. The results of the KMO test, which were above 0.80, indicate acceptable sampling adequacy and imply the appropriateness of factor analysis. It has been suggested that KMO values above 0.80 are considered “meritorious” while values above 0.90 are considered “marvellous” (17). The appropriateness of factor analysis was also supported by the results of Bartlett's test of sphericity. The values of Cronbach's alpha and composite reliability of the scales were found to be sufficient and internal consistencies met the adequacy criteria (18). The initial eigenvalues above one for both the impact and coping scales also suggest that all items fit into the theoretical construct. The results of this study show that the COVID-19 impact scale is structurally valid, as evidenced by factor analysis results with three robust components: well-being and lifestyle, psychological, and employment-related. Likewise, the coping scale with its three components, i.e., mindfulness practise, physical coping, and help seeking, is also valid and reliable. Therefore, we believe that the impact and coping scales have the potential to be used as measurement tools in future studies.

The results of the survey show that the implementation of containment during COVID-19 resulted in a prominent impact on psychological aspects. Most of the participants in this study reported constant fear of coronavirus infection, similarly reported in many other countries around the world (19–21). A high prevalence of a feeling of restlessness or irritability due to being under confinement was reported by our study participants, also similarly reported in other countries (22, 23); this points towards the considerable detrimental effects of COVID-19 containment measures on emotional and psychological health consequences. In this study, measures to contain the outbreak also led to the disruption of well-being and lifestyle, with many participants reporting a reduction in their overall happiness, engaging in unhealthy eating habits, disruption in sleep quality, and deterioration of overall health. It is recommended to limit COVID-19 impacts by maintaining a healthy lifestyle (e.g., exercising, eating healthy and at regular times, getting enough sleep, avoiding drug and alcohol use), planning a daily routine, getting involved in pleasant activities, and connecting with trusted others to share concerns and feelings (24–26), and should be encouraged to the general public in Malaysia.

It is important to highlight that the insurmountable social isolation, loneliness, boredom, financial hardship, and other pandemic-related bereavements associated with prolonged home confinement and lockdown during the COVID-19 pandemic have been reported to result in a surge of behavioural addiction (27). A surge in the sale of alcohol and use of tobacco and electronic cigarettes has been observed during the lockdown period in Western countries (27–30). Similarly reported in this study, the consumption of alcohol and tobacco were evident. It has been suggested that a strong support system integrating family, society, healthcare providers, and government and legislative bodies is needed to provide support and treatment to people with substance use disorders, as well as prevention by limiting access to controlled substances; these are all important to tackle behavioural addiction and promote addiction-free living during the pandemic (27). Others addictive behaviours associated with the use of information and communications technology (ICT) during self-isolation in the pandemic, which were not assessed in this study, include gambling, video gaming, TV series watching, problematic social media use, watching pornography, or surfing the internet. These activities are often used to reduce stress and anxiety and/or to alleviate a depressed mood or boredom, and also warrant further investigation in future studies (31).

The MCO in Malaysia came into force on 18 March and ended on 8 June 2020, where subsequently the country moved into more lenient movement control orders. The near 3 months of strict movement control and shutdown of many businesses resulted in striking, negative employment-related consequences. In this study, economic consequences were evident by salary reduction and income losses in a short 3-month period, demonstrating the devastating effect of containment during the epidemic. It is a top priority for the country to plan for effective strategies to support affected households, particularly lower-income groups, in preparation for future pandemic containment. To date, as of the end of August, the pandemic is far from over. Although the country has eased containment measures, with almost all economic sectors resuming operations subject to compliance with recommended practises by the World Health Organisation (WHO), COVID-19 infections continue with single- or double-digit cases reported every day. The relaxation of containment measures after quarantine poses a COVID-19 re-emergence risk, as seen in the recent resurgence in COVID-19 cases in Japan and Australia (32–35). Hence, there is a need for campaigns to keep the public alert to the risks of new epidemics, the need for continuous personal protective behaviours and social distancing, and most importantly to be mentally prepared for the possibility of the reinforcement of outbreak containment. Additionally, setting up mental health and psychosocial support in disaster situations should a priority in terms of preparedness of the resurgence of COVID-19 or other pandemics (22).

Findings on coping showed that mindfulness practise followed by physical coping strategies were the most common practises used by the study participants during home confinement. Of note, during the MCO period, the Ministry of Health of Malaysia published well-described guidelines on mental health and psychosocial support in COVID-19 (36, 37). The Ministry of Health of Malaysia and other non-government organisations also set up free mental health hotlines to ease COVID-19 lockdown. High hotline usage was reported high during the MCO period in Malaysia (38, 39). Nonetheless, in this study, only a small proportion reported seeking professional help or reached out for hotlines for coping. This perhaps can be explained by our study participants not being severely affected by the COVID-19 pandemic, making them less likely to utilise hotlines or seek professional help. This is further strengthened by the results of the PLS-SEM demonstrating that help-seeking coping was associated with higher psychological, well-being and lifestyle, and employment impacts.

It is worth mentioning that mindfulness coping was inversely associated with all the three dimensions of impact, with significant associations observed in well-being and lifestyle and employment-related impact. These findings perhaps imply that the impact was decreased as a result of the practise of mindfulness. Numerous studies have reported that mindfulness practise brings about various positive psychological effects, including increased subjective well-being, reduced psychological symptoms and emotional reactivity, and improved behavioural regulation (40). Mindfulness-based e-mental health interventions have been recently reported as an innovative and useful approach to confront the mental health aspects of the COVID-19 pandemic and to support psychologically burdened people (41). Another possible explanation could be that the people who practise mindfulness coping were those who were less impacted in all three dimensions of impact. Due to the cross-sectional design, we cannot determine whether the associations observed are causally related or the potential direction of any effects. With regard to physical coping, however, this study found no significant association between physical coping and all three dimensions of impact. Further studies are warranted to determine the association between physical coping and the impact of quarantine and isolation to contain the COVID-19 pandemic.

It has been postulated that the pandemic's economic consequences may disproportionately affect socially disadvantaged people in society (42), as was also evident in this study. Our finding of an inverse association between income level and all three dimensions of impact in the PLS-SEM infers that people with higher financial means were more likely to experience adverse consequences to confinement during the COVID-19 pandemic. Furthermore, education level was also found to be inversely associated with well-being and lifestyle. Both findings imply that socioeconomic status influenced the impact of containment during the COVID-19 epidemic. Hence, the provision of psychological support and coping, as well as economic subsidies are essential for lower socio-economic groups. COVID-19 experience and health status were found to have no significant influence on impact in the PLS-SEM.

It is worth noting a few limitations of the present study, particularly concerning the study design and data collection method. Firstly, due to the cross-sectional design, the directionality of the association or the causal relationship between coping and impact could not be established; however, the findings provide a basis for acquiring and testing this causal hypothesis. Due to various resource limitations during the disease crisis and movement restriction in Malaysia, convenience sampling using an online web-based survey via a social media platform may lead to selection bias, as reflected in the large sample of females, people of higher education, and the majority being from the central region. Hence, lower-educated people and people living in remote areas were under-represented. Despite the lack of general population representativeness, which may affect the generalisability of our findings, the current study provides useful first-hand information on the impact on the public during MCO and their coping behaviours. It is also worth pointing out that the MCO period was 18 March to 12 May; however, our survey period was 10 May to 7 July 2020; this study queried participants about impact and coping during the MCO period, so may be subjected to recall bias. In view of the above limitations, the findings of this study should be interpreted with caution. It is also important to note that the total variance explained by the by the PLS-SEM models seems small (5.5–9.5%), therefore caution must be taken when interpreting the results. Despite the limitations mentioned above, this study provides importance insights into the assessment of impact of containment and coping behaviours during the pandemic of infectious disease. Future research should emphasise on conducting quantitative content validation to improve the impact and coping scales developed in this study.

## Conclusion

Our study found that the developed impact and coping measurements have adequate validity and reliability and can be used in future research. All the constructs in the measurements have an acceptable level of internal consistency. The survey findings revealed that psychological impact was the most prominent, followed by impact on well-being and lifestyle. Mindfulness and physical coping were the most commonly used mechanisms in response to movement containment during the coronavirus pandemic. Exploring the relationship between coping and impact revealed that people who seek for health professional help were those with highest levels of impact in the psychological, well-being and lifestyle, and employment-related components. Coping through mindfulness practise was found to bring improvements in well-being and lifestyle, as well as employment-related impacts. Encouraging use of helplines and seeking professional help are essential in responding to the COVID-19 pandemic. Promoting mindfulness, coping, and resilience to the unpleasant impacts of quarantine is deemed necessary to face the resurgence of COVID-19 or future pandemics.

## Data Availability Statement

The raw data supporting the conclusions of this article will be made available by the authors, without undue reservation.

## Ethics Statement

The studies involving human participants were reviewed and approved by University of Malaya Research Ethics Committee (UM.TNC2/UMREC - 922). The participants provided their online informed consent to participate in this study.

## Author Contributions

LPW, KMT, YL, and ZH planned the study. LPW, HA, KMT, HYL, SA, PSKT, and MM obtained the data. LPW, HA, and MD performed the data analysis and data summarization. LPW drafted the manuscript. All authors critically reviewed the manuscript, gave final approval of the version to be published, agreed on the journal to which the article has been submitted, and agreed to be accountable for all aspects of the work.

## Funding

This study received financial supports from Ministry of Education Malaysia under Long Term Research Grant Scheme (LRGS MYRUN Phase 1: LRGS MYRUN/F1/01/2018) and Special Projects of the Central Government Guiding Local Science and Technology Development (No. 2021L3018), China.

## Conflict of Interest

The authors declare that the research was conducted in the absence of any commercial or financial relationships that could be construed as a potential conflict of interest.

## Publisher's Note

All claims expressed in this article are solely those of the authors and do not necessarily represent those of their affiliated organizations, or those of the publisher, the editors and the reviewers. Any product that may be evaluated in this article, or claim that may be made by its manufacturer, is not guaranteed or endorsed by the publisher.
