# Supplementary material for: Assessment of Impact of Containment During the COVID-19 Epidemic and Coping Behaviours Using Newly Developed Assessment Tools
Source: Front Public Health. 2021 Dec 22;9:787672. doi: 10.3389/fpubh.2021.787672 (PMC8728738; doi:10.3389/fpubh.2021.787672)
Supplement: Supplementary file 1 [file Data_Sheet_1.DOCX]

**Appendix 1 Impact and Coping Questions**

**Impact**

| Items | **Not at all** | **Moderate** | **Extremely** |
| --- | --- | --- | --- |
| D1. Drop in earning (income loss) | **0** | **1** | **2** |
| D2. Suffer pay cut | **0** | **1** | **2** |
| D3. Lost in work productivity | **0** | **1** | **2** |
| D4. Overall health deteriorates | **0** | **1** | **2** |
| D5. Overall level of happiness reduced | **0** | **1** | **2** |
| D6. Strained relationship with loved ones/family members in the household | **0** | **1** | **2** |
| D7. Constantly fear of being infected with COVID-19 | **0** | **1** | **2** |
| D8. Disruption in sleep quality | **0** | **1** | **2** |
| D10. Unhealthy eating habit (over eating, or indulge in unhealthy food) | **0** | **1** | **2** |
| D11. Restless/irritable for unable to attend worship services, rituals or prayers | **0** | **1** | **2** |
| D12. Restless/irritable due to separation (from loved ones/family members) | **0** | **1** | **2** |
| D13. Restless/irritable for unable to perform my usual outdoor routines and activities | **0** | **1** | **2** |

**Coping behavior**

| Items | **Not at all** | **Sometimes** | **Most of the times** |
| --- | --- | --- | --- |
| E1. Tried to think positive | **0** | **1** | **2** |
| E2. Tried to keep busy | **0** | **1** | **2** |
| E3. Tried to keep negative feeling under control | **0** | **1** | **2** |
| E4. Tried to keep my life as normal as possible and not to let it interfere | **0** | **1** | **2** |
| E5. Used relaxation technique (listening to music, painting, meditate, yoga etc.) | **0** | **1** | **2** |
| E6. Talked the problem over with others | **0** | **1** | **2** |
| E7. Take breaks, get plenty of rest or sleep | **0** | **1** | **2** |
| E8. Engage in physical exercise | **0** | **1** | **2** |
| E9. Seek professional help (counsellor, doctor) | **0** | **1** | **2** |
| E10. Reach out to COVID-19 hotlines | **0** | **1** | **2** |
